# Differences in Forage-Acquisition and Fungal Enzyme Activity Contribute to Niche Segregation in Panamanian Leaf-Cutting Ants

**DOI:** 10.1371/journal.pone.0094284

**Published:** 2014-04-09

**Authors:** Pepijn W. Kooij, Joanito Liberti, Konstantinos Giampoudakis, Morten Schiøtt, Jacobus J. Boomsma

**Affiliations:** Centre for Social Evolution, Department of Biology, University of Copenhagen, Copenhagen, Denmark; Arizona State University, United States of America

## Abstract

The genera *Atta* and *Acromyrmex* are often grouped as leaf-cutting ants for pest management assessments and ecological surveys, although their mature colony sizes and foraging niches may differ substantially. Few studies have addressed such interspecific differences at the same site, which prompted us to conduct a comparative study across six sympatric leaf-cutting ant species in Central Panama. We show that foraging rates during the transition between dry and wet season differ about 60 fold between genera, but are relatively constant across species within genera. These differences appear to match overall differences in colony size, especially when *Atta* workers that return to their nests without leaves are assumed to carry liquid food. We confirm that Panamanian *Atta* specialize primarily on tree-leaves whereas *Acromyrmex* focus on collecting flowers and herbal leaves and that species within genera are similar in these overall foraging strategies. Species within genera tended to be spaced out over the three habitat categories that we distinguished (forest, forest edge, open grassland), but each of these habitats normally had only a single predominant *Atta* and *Acromyrmex* species. We measured activities of twelve fungus garden decomposition enzymes, belonging to the amylases, cellulases, hemicellulases, pectinases and proteinases, and show that average enzyme activity per unit of fungal mass in *Atta* gardens is lower than in *Acromyrmex* gardens. Expression profiles of fungal enzymes in *Atta* also appeared to be more specialized than in *Acromyrmex*, possibly reflecting variation in forage material. Our results suggest that species- and genus-level identities of leaf-cutting ants and habitat-specific foraging profiles may give predictable differences in the expression of fungal genes coding for decomposition enzymes.

## Introduction

Maximizing the acquisition of high quality food under varying ecological conditions is expected to be under continuous natural selection. This notion has inspired many studies addressing optimal foraging strategies [1] and the extent to which related species realize niche segregation [2] and character displacement [3] to avoid interspecific competition. These processes often lead to (sub)habitat segregation [4]–[6] or food specialization, but few comparative studies have focused on generalist insect herbivores because it often remains unclear whether specialization within generalist strategies does in fact occur and what the decisive axes are along which niches and habitats may segregate [7], [8]. This question is particularly relevant for social insects, as they are central place foragers and often have a large impact on their surrounding communities. For wood eating termites that live in their food, pest management agencies will automatically accumulate comparative data on habitat and niche segregation among species and genera [9], [10], but such comparative studies have remained rare in the leaf-cutting ants.

*Atta* [Fabricius, 1804] and *Acromyrmex* [Mayr, 1865] leaf-cutting ants originated between 8 and 12 million years ago as the most specialized crown-group of the fungus growing ants (Attini [Emery, 1913]) [11]. Their extant distribution ranges from warm-temperate South America up to the southern regions of the United States [12]–[14]. Throughout this range these ants are important (often dominant) herbivores and significant accelerators of nitrogen and phosphorus cycling [15]. They decompose the harvested live plant material through the mutualistic services provided by their fungus-garden symbiont *Leucoagaricus gongylophorus* [Singer, 1986], which feeds the ants in exchange for the plant substrate provided [16]. Weber [14] estimated that ca. two kg of fresh plant material is needed to build one fungus garden in an *Atta cephalotes* [Linnaeus, 1758] colony and that almost 6000 kg of fresh vegetation had been processed by the collective fungus gardens of a 6.5 year old colony of *Atta sexdens* [Linnaeus, 1758].

Many species of leaf-cutting ants are considered pests in agricultural and urban areas [17]. For economic damage assessments, the genera *Atta* and *Acromyrmex* are often considered indiscriminately, in spite of large differences in colony size [14], [18], degree of worker polymorphism [18]–[20], fungus garden enzyme activity [21], and foraging behavior [14], [19], [22], [23]. For example, Cherrett [24] showed that forage material of an *Atta cephalotes* colony in Guyana consisted mostly of leaves with flowers as a distinct minority class, similar to a later studied colony of *Atta colombica* [Guérin-Méneville, 1844] in Panama [25], whereas forage of Costa Rican *Acromyrmex octospinosus* [Reich, 1793], *Acromyrmex coronatus* [Fabricius, 1804] and *Acromyrmex volcanus* [Wheeler, 1937] is known to consist of leaves, flowers and some fruit fragments [26]. However, to our knowledge no studies have been done to quantify differences of this kind simultaneously at the same site for an entire local guild of leaf-cutting ants. This implies that habitat-specificity, foraging efficiency, and leaf processing in fungus gardens have not been compared with formal statistical analyses.

So far, 40 species of leaf-cutting ants have been described [11], [18], and they have all been hypothesized to rear the same polymorphic species of *L. gongylophorus* as fungal symbiont [27], despite the enormous distribution range mentioned above [12], [28] and the highly variable habitats and forage availabilities [18], [22], [24], [28]–[32]. A recent study [33] has indicated that the extant *L. gongylophorus* species is only 2–3 million years old, inferring that it must have swept through all leaf-cutting ant species while replacing the original fungus garden symbiont(s) that they had retained after coming into existence 8–12 million years ago. Other recent studies have shown that the *L. gongylophorus* fungus garden symbiont is highly plastic in its enzymatic responses to the various leaf-substrates that the ants deposit on their fungus gardens [21], [34], suggesting that forage type may systematically affect the expression of decomposition enzymes.

The objective of our study was to design a sampling scheme that allows the key characteristics of forage acquisition and processing to be compared across an entire guild of leaf-cutting ants. To achieve that goal, we quantified the diversity of forage material and the absolute and relative foraging rates for six sympatric leaf-cutting ant species in the month of May, around the start of the rainy season, in Gamboa, Panama: *Atta cephalotes*, *Atta sexdens*, *Atta colombica*, *Acromyrmex echinatior* [Schultz, Bekkevold & Boomsma, 1998], *Acromyrmex octospinosus*, and *Acromyrmex volcanus*. We supplemented our comparative data on foraging rates and substrate diversity with field measurements on the activity of extracellular enzymes in the fungus gardens maintained by the six leaf-cutting ant species to assess whether foraging preferences might be related to specific garden processing activities.

## Materials and Methods

### Ant Foraging Behavior

In May 2011 we located 9–11 foraging trails each for five of the six ant species (Table 1), always <30 m from the nest for *Atta* and <5 m from the nest for *Acromyrmex*. The sixth species, *Acromyrmex volcanus*, was so rare that only one trail was found. We observed *Acromyrmex* trails for 15 to 30 min and *Atta* trails for 2 min (or 4 times 0.5 min when trails were very busy) to obtain comparable data when counting ants that passed an imaginary line perpendicular to the trail. We replicated observations by sampling either trails of different colonies or multiple trails of the same colony going in different directions so they could be considered as independent samples of foraging habitat (Table S1). Diversity of forage material was classified in six categories: (parts of) flowers, (pieces of) fruit, herbaceous leaves, tree-leaves, other material (always rare), and ants carrying nothing on their way back to the colony. When in doubt, we verified the origin of forage particles by backtracking the trail to the source. Observations were repeated across parts of the day (morning 9 AM–12 PM, afternoon 12 PM–5 PM, evening in the dark 10 PM–11 PM) and compared statistically to see whether this made any difference. The Smithsonian Tropical Research Institute (STRI), Panama, and the Autoridad Nacional del Ambiente y el Mar (ANAM) provided research permits to sample ant colonies, logistic help and facilities to work in Gamboa.

**Table 1:** Differences in foraging rate between loaded and unloaded foragers.

Genus	Species	Trails	Observation	Total ants	Loaded ants per hour		Unloaded ants per hour	
*Acromyrmex*	*echinatior*	10 (10)	285 min	265	80±14	58±10	0±0	0±0
	*octospinosus*	10 (10)	285 min	458		102±26		0±0
	*volcanus*	1 (1)	30 min	43		86		0
*Atta*	*cephalotes*	11 (6)	26 min	1519	3374±476	2740±456	1640±203	2408±373
	*colombica*	10 (7)	24.5 min	1724		5383±1003		1115±188
	*sexdens*	9 (7)	22 min	801		1173±447		1012±301

Differences in foraging rate between loaded and unloaded foragers of *Atta* and *Acromyrmex* species in Gamboa, Panama, with summary statistics on the number of trails observed per species (number of colonies in brackets), the total number of minutes of observation per species, the total number of ants counted while returning to their nests, and the foraging rates for loaded and unloaded returning workers: means (± SE) per genus and per species (see Table S1 for details).

Based on earlier field surveys at this mosaic landscape of secondary growth forest and suburban areas for a period of two decades the following generalizations of habitat differentiation [35] appear to apply in Gamboa: *Atta cephalotes* and *Acromyrmex volcanus* are forest canopy foragers, whereas *Acromyrmex octospinosus* forages on the forest-floor. *Atta colombica* occurs both in the forest (usually at lower elevations) and in moist open grassland habitats, while *Atta sexdens* and *Acromyrmex echinatior* prefer open and sunlit nesting habitats for foraging. The latter two species extend their distributions towards the Pacific coast where annual rainfall is less than in Gamboa and natural habitat resembles savannas rather than a mosaic of forest patches [12], [28], matching their preference for open habitat in Gamboa. We thus had some a-priori qualitative notions to work with and designed the present study to quantify them.

To assess rates of foraging, we expressed our records in numbers of workers on trails per hour, counting both workers carrying material back to the nest and those without. Data were log-transformed to approximately equalize variances and analyzed with R [36], using Linear Mixed-Effects Models (“lme”) [37] and generating p-values with General Linear Hypotheses (“glht”) in the package “multcomp” [38]. Proportional distributions of forage types (tree-leaves, herbaceous leaves, flowers, fruit, other) were analyzed with the same tests, with separate trails being considered as a random factor in both analyses. To test for heterogeneity across trails within species we created a sub dataset consisting of four *Atta* trails from two colonies (one *Atta colombica* and one *Atta cephalotes*) and compared the foraging category scores between trails and species (with trails nested within species, and 0.5 min replicate observations for each trail). This was only possible for these *Atta* species, as we did not have replicate samples within single trails for *Acromyrmex* (Table S1).

Results for the proportional distribution of forage types were visualized using “heatmap.2” in the R package “gplots” [39]. Dendrograms were calculated with the “pvclust” package [40] using 1000000 bootstrap iterations. Final clustering plots were based on the overall similarities in mean proportions (p) between species and supplemented by estimates of the inverse Simpson Diversity index (D = 1/[Σp_i_^2^]) to allow an explicit analysis of the degree of evenness (high D) between the different forage or expressed enzyme categories across species and genera of leaf-cutting ants. The denominator of the index decreases when more categories (p) enter the equation, but when the number of categories is constant (as in our analyses) more even distributions will give lower sums in the denominator and thus higher values of D. For the purpose of our study, D therefore functions as an index of generalist foraging or equal enzyme expression, so that high values (low Σp_i_^2^) indicate that all categories are important and low values (high Σp_i_^2^) indicate specialization either on a subset of forage categories or on a subset of expressed enzymes that were most active.

### AZCL Enzyme Activity Assays

For each of the six ant species the garden enzyme profiles were analyzed for five different colonies, with the exception of *Acromyrmex volcanus* for which only one colony was available, but where we could add data for another colony obtained in the previous year by H. H. De Fine Licht (pers. com.). For each colony, fungus gardens were dug up, and about equal size fragments (ca 80 mg) from top, middle and bottom layers of fungus gardens were collected and immediately homogenized together to obtain representative average enzyme activity measures per colony. These measurements were performed using previously published methods, which are easily applicable in the field and give repeatable results [21], [34], [41]. In short, fungus garden material (ca. 240 mg) was crushed with a pestle in a 1.5 ml eppendorf-tube containing 1000 μl 0.05 M TRIS-HCl buffer (pH 7.0), vortexed immediately and then centrifuged for 15 minutes (15000 g) after which the supernatant was removed and applied immediately to each of 12 different assay-plates containing 0.1 g/L of the Azurine-Crosslinked (AZCL) substrates: amylose, arabinoxylan, barley β-glucan, casein, collagen, debranched arabinan, galactan, galactomannan, HE-cellulose, rhamnogalacturonan, xylan and xyloglucan that were chosen because they yielded positive enzyme activities in an earlier study [21].

The assay-plates of 6 cm diameter were prepared separately for each substrate using an agarose medium (1% agarose, 23 mM phosphoric acid, 23 mM acetic acid, 23 mM boric acid), and pH adjusted according to the manufacturer’s description (Megazyme, Bray, Ireland). After the medium had solidified, round wells (area of ca. 0.1 cm^2^) were made in each plate with a cut-off pipette tip and 12 μl of the supernatant was applied to each well in triplicate. After 22 hours of incubation at 25°C the plates were photographed and the area of the blue halo surrounding each well (a quantitative measure for the absolute amount of enzyme activity [21], [34]) was measured using the software program ImageJ ver. 1.43u for Macintosh. Enzyme activity measurements were grouped into categories based on which plant cell wall component is the main target of the enzymes ([42] and Megazyme, Bray, Ireland): amylases (measured with amylose), cellulases (measured with barley β-glucan and HE-cellulose), hemicellulases (measured with arabinoxylan, galactomannan, xylan and xyloglucan), pectinases (measured with debranched arabinan, galactan and rhamnogalacturonan), and proteases (measured with casein and collagen). We present these data grouped for five categories of enzymes, implying that each of these categories had data from 2–3 enzymes, except for amylases that were represented by only a single enzyme amylose.

The enzyme activity scores were analyzed using a General Linear Model in SAS, with “colony” nested within “species” and “species” nested within “genus”. Colony was then treated as having a random enzyme class activity, composed of specific enzyme activities nested within enzyme activity classes. This procedure implied that we had to omit amylase, because we only had a single substrate (amylose = starch) for testing activity and because starch is not a primary challenge in the degradation of plant material [34]. We also left out rare *Acromyrmex volcanus* and thus report original mean values for enzyme activity for all six species and ten enzyme classes, whereas statistics given in the text refer to the reduced data set of four enzyme classes and the gardens of five ant species. In our final analyses we combined the enzyme and foraging datasets and visualized patterns of association with the plot.PCA function after Principal Component Analysis (“PCA”) using the “FactoMineR” package [43].

## Results

The three *Atta* species had an average foraging rate of 5014±555 SE ants/h, with 3374±476 SE ants (67%) returning to the nest carrying forage material and 1640±203 returning unloaded, whereas the three *Acromyrmex* species had an average foraging rate of 80±14 SE ants/h (t_52.444_ = −19.347, p<0.0001) and no workers returning without forage (Table 1). Separate analyses, using the probability of a foraging trail having loaded ants, showed that the genus-level differences in loaded and unloaded returning foragers per hour were highly significant (χ^2^_1_ = 7.567, p<0.01), but that the differences in loaded returning workers between species within genera were not significant (χ^2^_4_ = 1.257, p = 0.87).

**Figure 1:**
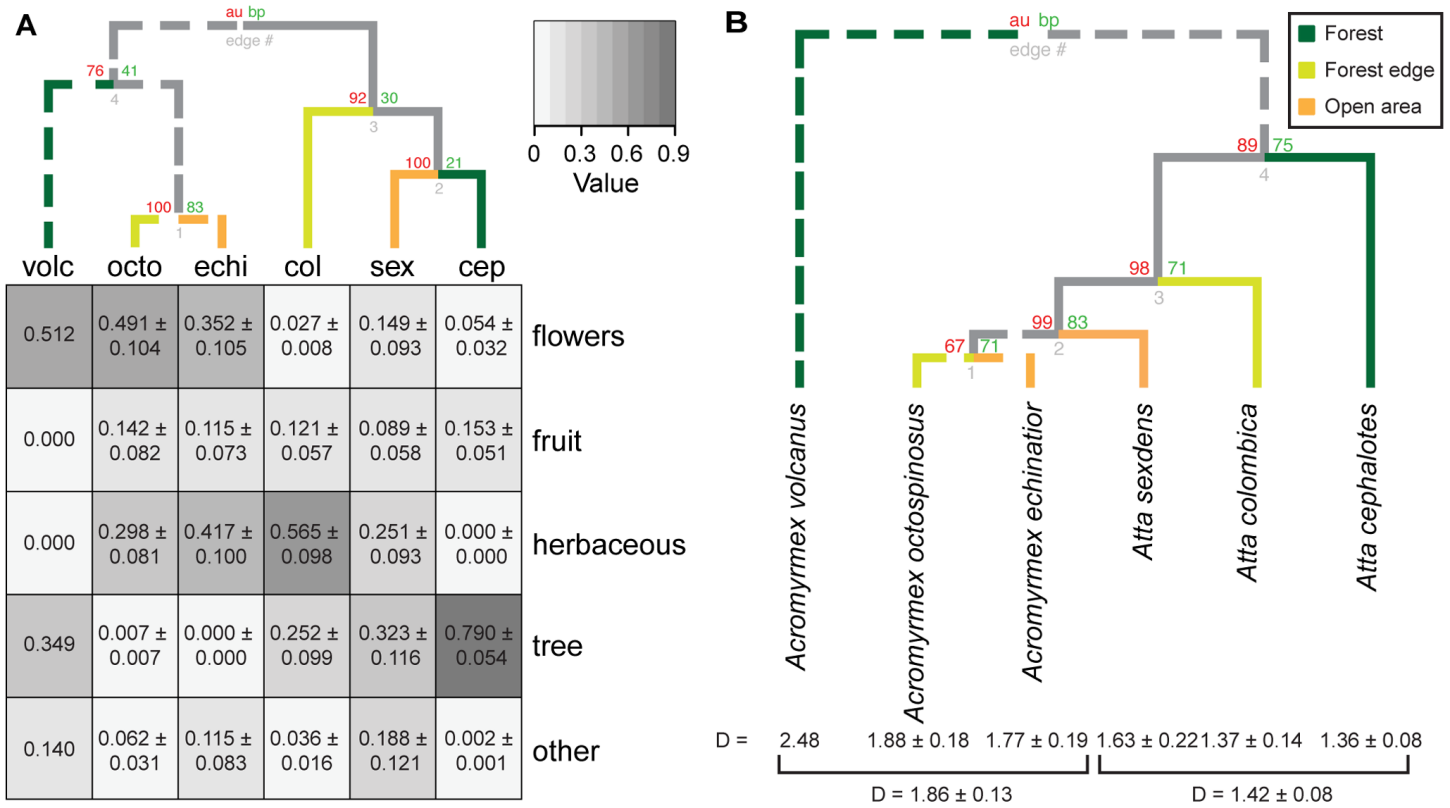
Differences in forage diversity. Differences in forage diversity between leaf-cutting ant species (nested within genera), using solid lines for *Atta* and dotted lines for *Acromyrmex*, and with typical foraging habitat indicated with dark green (forest), yellow (forest edge), and orange (open sunlit areas): (A) Heatmap showing differences between species and genera in the use of forage categories, with numbers representing mean proportions ±SE of the forage types. Darker colors indicate higher mean acquisition proportions, with the top-dendrogram illustrating similarities between species/genera across means of the five forage categories (vertical axis). Ant species names are given as abbreviations (volc, octo, echi, col, sex, cep). (B) Dendrogram based on the Inverse Simpson Diversity Index of the five forage categories, indicating the degree of evenness across foraging categories (numbers below the branches are mean D-values ±SE per species and means per genus), showing that *Acromyrmex* has a broader (more even) spectrum (D = 1.86±0.08 SE) of forage material than *Atta* (D = 1.42±0.13 SE; F_1,49_ = 5.435, p<0.05). Numbers above the branch nodes represent Approximately Unbiased p-values (AU, red) and Bootstrap Probability values (BP, green).

Across genera, *Atta* foragers harvested significantly more tree-leaves than *Acromyrmex* workers (z = 6.420, p<0.0001), while *Acromyrmex* foragers collected significantly more (pieces of) flowers than *Atta* workers (z = −4.894, p<0.0001). However, there were also differences in the most abundant forage category within genera. All *Acromyrmex* species preferred some combination of flowers and herbaceous leaves, but *Acromyrmex volcanus* was more flower-biased and *Acromyrmex echinatior* more herbaceous-leaves-biased (Figure 1A). Similarly, while *Atta cephalotes* primarily harvested tree-leaves (*cephalotes* vs *sexdens*, z = 4.987, p<0.001; *cephalotes* vs *colombica*, z = 6.782, p<0.0001), *Atta colombica* brought in more herbaceous leaves (*cephalotes* vs *colombica*, z = −7.109, p<0.0001), and *Atta sexdens* had approximately equal shares of all forage categories (Figure 1A), which confirmed earlier findings by De Vasconcelos [44].

We validated the statistical independence of our trail samples, using a subset of two *Atta* colonies (one *Atta colombica* and one *Atta cephalotes*), for which we had four replicated samples of the same trails (0.5 min each) and two separate trails per colony (Table S1). This recovered our earlier result that the two *Atta* species have different fractions of forage categories (F_4,60_ = 89.48, p<0.0001), but also showed that different trails of the same colony yielded similar results in spite of covering non-overlapping fractions of the colony’s foraging habitat (F_8,60_ = 0.75, p = 0.65). Further ANOVA showed that frequencies of forage types between the different times of the day were significantly different for *Atta* species (F_8,218_ = 4.451, p = 0.0001), and a post-hoc test indicated this was due to a higher share of herbaceous leaves in the afternoon compared to the evening (z = −3.929, p = 0.009). *Acromyrmex* species did not show any activity in the dark (evening) and frequencies of forage types between morning and afternoon observations were not different (F_4,76_ = 1.457, p = 0.224).

Ranking the six species according to the diversity of forage material (Figure 1B) gave no significant difference between species within genera in evenness of forage category use (F_5,45_ = 1.776, p = 0.137), but the pooled *Atta* species had a lower evenness in forage category use (D = 1.42±0.08 SE) than the pooled *Acromyrmex* species (D = 1.86±0.13 SE; F_1,49_ = 5.435, p = 0.024). After excluding *Acromyrmex volcanus* where sample size was very small and two forage categories completely missing, the evenness trends in Figure 1B corresponded fairly well with the relative proportional forage acquisition data in Figure 1A with, from left to right, a clearly increasing trend in tree-leaf use, a decreasing trend in the use of flowers, and hump-shaped trends in the use of herbaceous leaves and fruit. These inferences were supported by moderately high overall Approximately Unbiased (AU) p-values and Bootstrap Probability (BP) values (Figure 1). As night foraging tended to decrease the acquisition of herbaceous leaves by *Atta* species, we probably underestimated the difference in dependence on tree leaves between *Acromyrmex* and *Atta* because we obtained most of our *Atta* observations at daytime.

**Figure 2:**
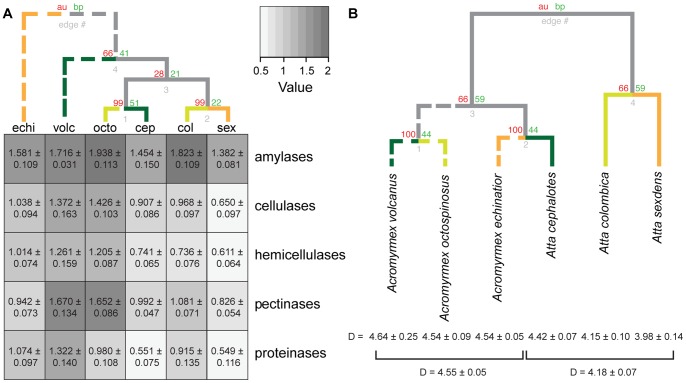
Differences in fungus garden enzyme activity. Differences in fungus garden enzyme activity between species grouped as in Figure 1 with solid lines for *Atta* and dotted lines for *Acromyrmex*, and with dark green, yellow and orange indicating the same habitat categories: (A) Heatmap showing differences between species and genera in fungus garden activity of enzyme classes, expressed as mean area in cm^2^±SE of colored halos on AZCL plates across all assays for enzymes belonging to the amylases (1), cellulases (2), hemicellulases (4), pectinases (3) and proteinases (2). Darker colors in the heatmap indicate higher mean activities, and the top-dendrogram illustrates similarities between species across all means for the five groups of enzymes, estimated by “pvclust” with 1000000 bootstraps. (B) Dendrogram based on the inverse Simpson Diversity Index of proportional enzyme activity showing that *Acromyrmex* fungus gardens have more even secretions across enzyme categories (D = 4.55±0.05 SE) than *Atta* (D = 4.18±0.07 SE, F_1,52_ = 15.006, p<0.0001). Numbers above the branch nodes represent Approximately Unbiased p-values (AU, red) and Bootstrap Probability values (BP, green).

In absolute quantities, fungus gardens of *Acromyrmex* showed a higher overall enzyme activity than gardens of *Atta* (F_1,20_ = 8.54, p<0.01) (Figure 2A), but species within genera did not show significant additional differences (F_3,20_ = 1.32, p = 0.29). To further investigate the differences in enzyme activity between the two genera, we decomposed the significant interaction term of ant genus and AZCL category (F_10,780_ = 4.95, p<0.0001). This revealed significant differences between *Acromyrmex* and *Atta* for all substrates except rhamnogalacturonan. The spectrum of relative enzyme activities, as expressed by the inverse Simpson indices (Figure 2B), showed that *Acromyrmex* species tend to have more evenly distributed enzyme activities (D = 4.55±0.05 SE) than *Atta* species who tend to specialize more on the expression of specific classes of enzymes (D = 4.18±0.07 SE; F_1,52_ = 15.006, p<0.0001). No significant differences were observed for the evenness of the enzyme activity spectra for species within genera (*Atta*: F_2,12_ = 1.618, p = 0.239; *Acromyrmex*: F_2,9_ = 0.111, p = 0.896).

**Figure 3:**
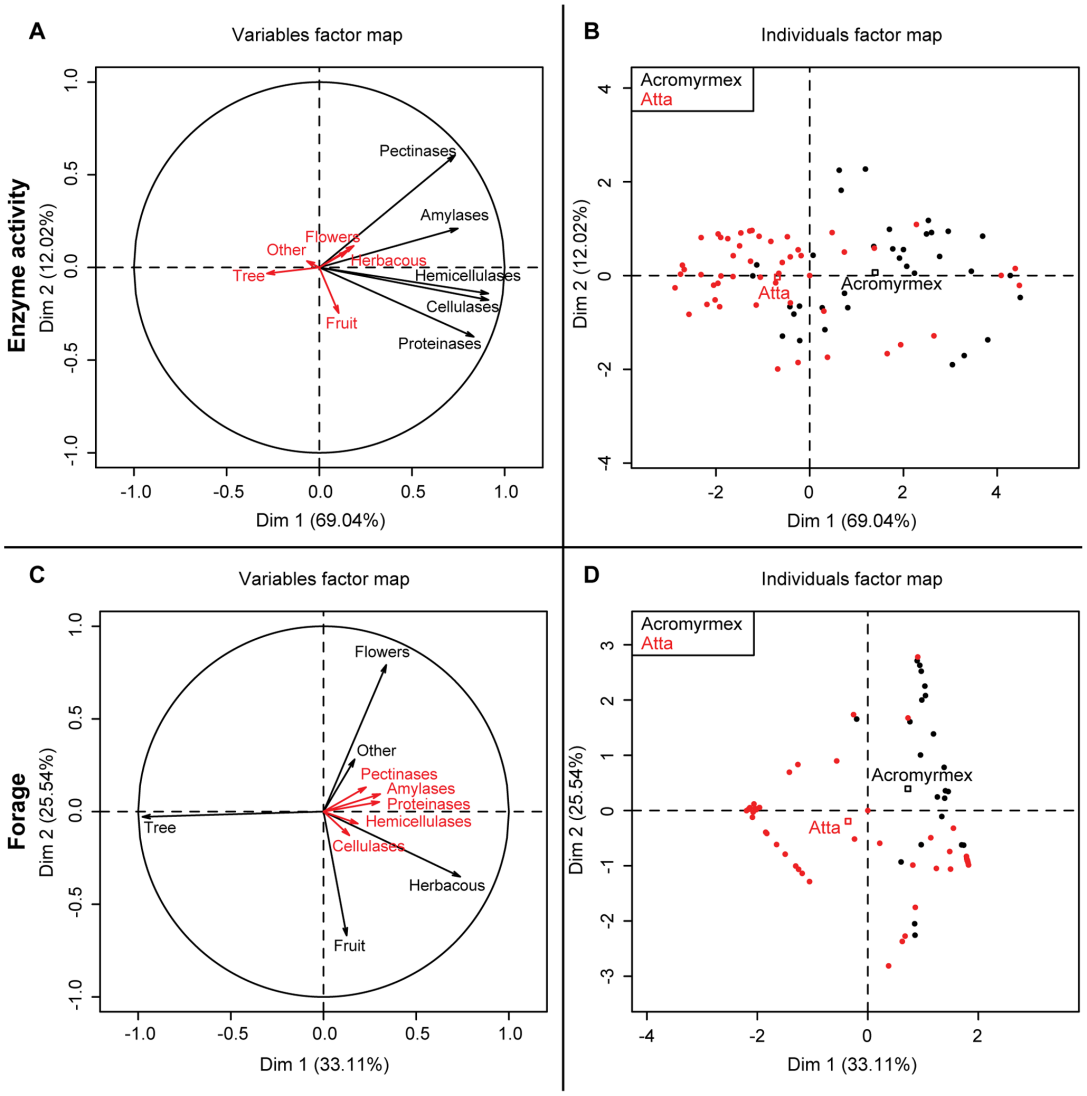
Principal Component Analyses on forage diversity and enzyme activity. Principal Component Analyses (PCA) using either the five enzyme groups of Figure 2 (A and B) or the five forage material categories of Figure 1 (C and D) as predictor variables (black arrows). Red arrows represent response vectors for forage material (A) or enzymes (C). The B and D panels complement the respective A and C panels by plotting PCA’s scores across the fungus garden measurements (B, 45 for *Atta* and 32 for *Acromyrmex*) the sampled ant trails (D, 30 for *Atta* and 21 for *Acromyrmex*; Table 1), largely separating the ant genera along the x-axes, confirming that *Atta* primarily focuses on tree-leaf material (compare panels C and D) and *Acromyrmex* on herbaceous leaves, flowers and (less pronounced) fruit. Comparison of the A and B panels illustrates that enzyme activity was generally higher in *Acromyrmex* (towards the right).

Comparative analyses (PCA), with either fungus garden enzyme expression as a predictor variable and forage diversity as a response variable (Figure 3A and B) or vice versa (Figure 3C and D), confirmed a separation between the genera *Atta* and *Acromyrmex* (Figure 3B and D). Taking the fungus garden enzyme activities as predictor variables produced a first axis explaining 69.04% of the variation and a second axis explaining 12.02% of the variation. The first axis corresponded to overall enzyme activity and illustrates that general fungus garden enzyme activity is lower towards the left (predominantly *Atta*) and higher towards the right (predominantly *Acromyrmex*) (Figure 3A and B), confirming the results given in Figure 2. The vertical axis reflects higher amounts of pectinases (positive scores) versus higher amounts of proteases (negative scores). This did not correspond in any obvious way with genus-level differences, but may be related to colony-level differences in the proportions of flowers and fruit in the forage (Figure 3A and B).

A similar pattern was obtained when the predictor and response variables were reversed (Figure 3C and D). The first axis (explaining 33.11% of the variation) illustrates a preference for tree-leaves (mostly *Atta*) towards the left and a preference for herbaceous leaves (mostly *Acromyrmex*) towards the right (Figure 3C and D). The second axis (explaining 25.54% of the variation) indicates higher intake of fruit (negative scores) in weak association with cellulases, and of flowers (positive scores) mostly in association with pectinases. Here the leaf-cutting genera are also separated to some extent (centroid squares) with *Acromyrmex* having higher preference for flowers and *Atta* for fruit, confirming the results depicted in Figures 1 and 2.

The PCA comparisons also revealed relations between forage preference and fungus garden enzyme expression, as both PCA analyses showed a negative relation between the extent of acquisition of tree-leaves and overall intensity of enzyme activity. To further test this we performed a Kendall’s rank test for correlation for all combinations of enzymes and forage material. This showed that this negative trend was significant for most enzymes: amylases (z = −3.185, p<0.01); pectinases (z = −2.608, p<0.01); proteases (z = −3.465, p<0.001), but not cellulases (z = −1.429, p = 0.153) and hemicellulases (z = −1.953, p = 0.051). The same analyses also found a negative correlation between fruit foraging and expression of amylases (z = −2.311, p<0.05) and positive correlations between foraging on herbaceous leaves and expression of amylases (z = 3.643, p<0.001), pectinases (z = 2.130, p<0.05) and proteases (z = 3.404, p<0.001) and between flower foraging and expression of the same enzymes: amylases (z = 2.712, p<0.01), pectinases (z = 3.066, p<0.01) and proteases (z = 3.769, p<0.001). Foraging on other materials was only (positively) correlated with the expression of pectinases (z = 2.366, p<0.05).

## Discussion

Although it is widely appreciated that *Atta* and *Acromyrmex* differ by more than two orders of magnitude in their scale of operations (see e.g. [14], [18], [26], [45]), systematic comparative studies similar to the present analyses have to our knowledge not been done. Although our snapshot results for the month of May cannot be generalized, we believe to have achieved our objective of demonstrating that larger scale studies like this can be done in principle. Our analyses illustrate that the statistical tools to analyze such data are available and can easily be expanded for use in more encompassing field surveys, with extra seasons, sampling sites, additional ant species, and new predictor variables within which genera, species, and colonies can be nested. In the sections below, we offer tentative interpretations and compare them with available literature.

### Genus-level Niche Segregation between *Atta* and *Acromyrmex*

Our results confirm that the two genera of leaf-cutting ants operate at different scales and show that their foraging niches are systematically different and that the enzymatic processing activities of fungus gardens appear to reflect these differences. The differences in foraging preferences quantified our intuitive expectations based on two decades of fieldwork in Gamboa, but the enzymatic activity differences were more substantial than we expected, because the two genera rear fungus-garden symbionts that belong to a single species *L. gongylophorus* [33]. This suggests that studies of phenotypic plasticity in enzyme gene expression will be worthwhile to enhance our understanding of the versatility of the leaf-cutting ant symbiosis. We will return to this in more detail below.

Our finding that average genus-specific foraging rates show a 42 fold difference in loaded-worker return rates and a 63 fold difference in total worker return rates per hour, seems to match the ca. two order of magnitude difference in colony size between *Atta* and *Acromyrmex*. The fact that the differences do not quite reach 100 fold [14], [18], [26], [46] may be due to our primary focus on the largest *Acromyrmex* colonies (smaller colonies have too little foraging activity making the type of sampling that we did less feasible), whereas our selection of *Atta* colonies mostly contained medium size colonies. It is also conceivable that the *Atta* workers that returned to their nests without carrying plant material may have had their crops filled with plant sap as suggested by Littledyke & Cherrett [47], Quinlan & Cherret [48] and Hölldobler & Wilson [19], but the present setup did not allow any measurements on this. This suggests that considering only loaded workers may underestimate foraging effort, and that larger scale comparative studies should include sampling of liquid food in the crops of returning foragers. In spite of these limitations, we will also return to tentative inferences on species- and genus-level niche segregation that our snapshot data for Gamboa appeared to allow.

### Garden Enzyme Activity and Forage Material – is there a Connection?

It has long been known that the fungus is a major producer of enzymes for the decomposition of plant material that leaf-cutting ant foragers provide, and recent work has shown that these decomposition services are supplemented by several other microorganisms that live in attine gardens [49], [50]. Other recent studies have emphasized that the expression of enzymes can be remarkably plastic and substrate dependent [21], [34]. This is consistent with earlier notions that there are active feedback loops between forager supply and symbiont demand, such that foragers may discard some forage material under specific conditions where its excess processing would not be optimal [51].

Our present results quantify the notion that *Atta* and *Acromyrmex* represent ecologically distinct ant genera, both with regard to forage acquisition/diversity and garden enzyme activity/diversity. We acknowledge that this may not necessarily apply in other Latin American regions and that our results thus make no predictions about the extent to which, for example, grass-cutting *Atta* and *Acromyrmex* should partition their foraging habitats on the Argentinean pampas. However, the sampling schemes and analyses reported here can also be applied in other habitats, so that any hypothesis suggesting that similar niche partitioning rules could apply also there can be tested and if need be rejected. The genus-level distinction in garden enzyme activity/diversity appears consistent with recent other results showing that proteomes differ between sympatric fungus gardens of *Acromyrmex echinatior* and *Atta cephalotes* [52]. Such functional differences between *Atta* and *Acromyrmex* cultivars could possibly be due to species and/or genera rearing different lineages of the same fungal species, consistent with *Atta* and *Acromyrmex* in Gamboa rearing non-overlapping subclades when using rapidly evolving microsatellite or AFLP markers to characterize them [53].

It is remarkable that our results indicate that *Atta* gardens generally produced lower amounts of enzymes, even though these ants forage mostly on tree-leaves (Figure 3), which one would expect to be more demanding to decompose. It also appeared that *Atta* gardens tended to overproduce two classes of enzymes, cellulases and pectinases, in addition to amylases (Figure 2), whereas *Acromyrmex* gardens produced higher amounts of all enzyme categories. This suggests that *Atta* gardens may somehow extract necessary nutrients more efficiently, but further work will be needed to understand the details of these processes. An additional factor to consider in this context is that *Atta* colonies produce conspicuous waste heaps or underground compost chambers, whereas this is rare for *Acromyrmex* (J.J. Boomsma & P.W. Kooij pers. obs.). This is consistent with Panamanian *Atta* discarding a larger fraction of not fully degraded older fungus garden biomass than *Acromyrmex* [54], [55], perhaps because average enzyme activity per unit of fungus garden mass is lower and fresh tree leaves are more abundantly available than flower parts.

An earlier comparative study [21] has hypothesized that *Atta* species focus on the rapid degradation of starch and proteins, but discard fungus garden material before most of the cellulose and hemicellulose is degraded. This is consistent with other recent studies showing that high amounts of cellulose and hemicellulose are still present in the bottom layer of fungus gardens [50], [56], [57] and that only cellulases from *L. gongylophorus* remain highly active in this bottom layer [52], [58]. The larger scale and more wasteful substrate processing practiced by *Atta* may thus leave more substantial niches for additional bacterial and/or yeast [49] decomposition, similar to the domestication of specialized gut bacteria in large ungulates [59] that rely on residues of leaf-material that were hard to digest even for ruminants. Focused comparative transcriptomics to investigate conditional gene expression in fungus gardens of the two leaf-cutting ant genera could shed further light on possible differences of this kind and metagenome sequencing could identify the microbial communities involved, similar to an earlier yeast study on the fungus gardens of *Acromyrmex* and *Atta* [49].

### Niche Partitioning in Panamanian *Atta* and *Acromyrmex*

The data provided in our study are a snapshot of year-round foraging, which is known to vary across the seasons [25]. This implies that we cannot be sure that sampling in other seasons or at other sites would have yielded similar results. However, we note that the five forage categories that we distinguished are very general and likely to be available throughout the year and that medium-size colonies of *Atta* and large colonies of *Acromyrmex* are unlikely to move over substantial distances (but see [60]), so their central place for foraging would tend to cover the same (sub) habitat over time. In spite of these caveats, our study shows that genus- and species-level differences across leaf-cutting ants can be quantified with the statistical tools we developed during this study.

The results of our study suggest that direct competition for forage material between the two genera of leaf-cutting ants is likely to remain limited, because *Atta* and *Acromyrmex* species target rather different types of forage, in spite of some overlap consistent with earlier reports that mostly report allopatrically collected data [18], [22], [24], [28]–[30], [32]. The correlations between garden enzyme activity and genus-level difference in forage use that we uncovered for the Gamboa community of leaf-cutting ants may be reinforced or supplemented by differences in salivary gland secretions between the two ant genera [61], a variable we were unable to measure. However, comparisons at the species level suggested that both *Atta* and *Acromyrmex* species tend to have habitats that are largely mutually exclusive, with *Acromyrmex volcanus* and *Atta cephalotes* foraging in the canopy, *Acromyrmex octospinosus* and (somewhat less specifically) *Atta colombica* foraging on the forest floor, and *Acromyrmex echinatior* and *Atta sexdens* foraging in the open landscape. Although it is possible that these differences are less pronounced in other seasons or sites, these results seem consistent with ecological theory predicting that interspecific competition is more pronounced when species are more similar, so that habitat partitioning may evolve [4]–[6].

The only case in which habitat segregation was somewhat less pronounced was between *Atta sexdens* and *Atta colombica*, which often overlapped in park-like and man-made habitats. Although there is a clear gradient across the isthmus of Panama, *Atta sexdens* is the dominant *Atta* species along the Pacific coast and becomes less abundant towards Gamboa in central Panama, whereas the pattern is opposite for *Atta colombica* [28]. It is interesting that these are the only two species for which we once observed active avoidance behavior on neighboring trails, i.e. trails of a colony stopping ca. one meter from the trail of another colony (P.W. Kooij, pers. obs.), behavior expected for all *Atta* spp. when foraging territories overlap. For the two common Panamanian *Acromyrmex* species, of which our research group has dug up ca. 500 colonies over the last two decades, habitat segregation (forest for *A. octospinosus* and open grassland areas for *A. echinatior*) is so pronounced that they will rarely encounter each other, similar to what is seen in Costa Rica [62]. In this way mature colonies of these species are unlikely to compete for the same type of plant forage. As far as we are aware distributions of incipient (founding) colonies are similar to those of mature colonies in Gamboa, but this is harder to quantify.

## Supporting Information

Table S1 **Sample sizes for each colony.**
(XLSX)

## References

[1] Stephens DW, Krebs JR (1986) Foraging theory. Princeton: Princeton University Press. 247 pp.

[2] Rohde K (2006) Nonequilibrium ecology. Cambridge: Cambridge University Press. 236 pp.

[3] Dayan T, Simberloff D (2005) Ecological and community-wide character displacement: the next generation. Ecol Lett 8: 875–894 10.1111/j.1461-0248.2005.00791.x

[4] Florencio M, Gómez-Rodríguez C, Serrano L, Díaz-Paniagua C (2013) Competitive exclusion and habitat segregation in seasonal macroinvertebrate assemblages in temporary ponds. Freshwater Sci 32: 650–662 10.1899/12-105.1

[5] Guo Z, Liu J, Lek S, Li Z, Ye S, et al (2012) Habitat segregation between two congeneric and introduced goby species. Fund Appl Limnol 181: 241–251 10.1127/1863-9135/2012/0397

[6] Ortega S (1987) Habitat segregation and temporal variation in some tropical intertidal populations. J Exp Mar Biol Ecol 113: 247–265.

[7] Ali JG, Agrawal AA (2012) Specialist versus generalist insect herbivores and plant defense. Trends Plant Sci 17: 293–302 10.1016/j.tplants.2012.02.006 22425020

[8] Bernays EA, Minkenberg O (1997) Insect herbivores: different reasons for being a generalist. Ecology 78: 1157–1169.

[9] Houseman RM, Gold RE, Pawson BM (2001) Resource partitioning in two sympatric species of subterranean termites, *Reticulitermes flavipes* and *Reticulitermes hageni* (Isoptera: Rhinotermitidae). Environ Entomol 30: 673–685 10.1603/0046-225X-30.4.673

[10] Kambara K, Takematsu Y (2009) Field habitat selection of two coexisting species of *Reticulitermes*, *R. speratus* and *R. kanmonensis* (Isoptera, Rhinotermitidae). Sociobiology 54: 65–75.

[11] Schultz TR, Brady SG (2008) Major evolutionary transitions in ant agriculture. P Natl Acad Sci USA 105: 5435–5440 10.1073/pnas.0711024105 PMC2291119 18362345

[12] Farji-Brener AG (2000) Leaf-cutting ant nests in temperate environments: mounds, mound damages and nest mortality rate in *Acromyrmex lobicornis* . Stud Neotrop Fauna E 35: 131–138.

[13] Mayhé-Nunes AJ, Jaffe K (1998) On the biogeography of Attini (Hymenoptera: Formicidae). Ecotropicos 11: 45–54.

[14] Weber NA (1966) Fungus-growing ants. Science 153: 587–604. 17757227 10.1126/science.153.3736.587

[15] Fowler HG, Pagani MI, Da Silva OA, Forti LC, Da Silva VP, et al (1989) A pest is a pest is a pest? The dilemma of Neotropical leaf-cutting ants: keystone taxa of natural ecosystems. Environ Manage 13: 671–675.

[16] Bass M, Cherrett JM (1995) Fungal hyphae as a source of nutrients for the leaf-cutting ant *Atta sexdens* . Physiol Entomol 20: 1–6.

[17] Cherrett JM, Peregrine D (1976) A review of the status of leaf-cutting ants and their control. Ann Appl Biol 84: 124–128.

[18] Mehdiabadi NJ, Schultz TR (2010) Natural history and phylogeny of the fungus-farming ants (Hymenoptera: Formicidae: Myrmicinae: Attini). Myrmecol 13: 37–55.

[19] Hölldobler B, Wilson EO (2011) The leafcutter ants. New York, London: W. W. Norton & Company, Inc. 160 pp.

[20] Weber NA (1958) Evolution in fungus-growing ants. Proceedings of the Tenth International Congress of Entomology, Vol. 2: 459–473.

[21] De Fine Licht HH, Schiøtt M, Mueller UG, Boomsma JJ (2010) Evolutionary transitions in enzyme activity of ant fungus gardens. Evolution 64: 2055–2069 10.1111/j.1558-5646.2010.00948.x 20067517

[22] De Fine Licht HH, Boomsma JJ (2010) Forage collection, substrate preparation, and diet composition in fungus-growing ants. Ecol Entomol 35: 259–269 10.1111/j.1365-2311.2010.01193.x

[23] Weber NA (1972) The fungus-culturing behavior of ants. Am Zool 12: 577–587.

[24] Cherrett JM (1968) The foraging behaviour of *Atta cephalotes* L. (Hymenoptera, Formicidae) I. Foraging pattern and plant species attacked in tropical rain forest. J Anim Ecol 37: 387–403.

[25] Wirth R, Herz H, Ryel RJ, Beyschlag W, Hölldobler B (2003) Herbivory of leaf-cutting ants: a case study on *Atta colombica* in the tropical rainforest of Panama. Berlin, Heidelberg, New York: Springer-Verlag. 230 pp.

[26] Wetterer JK (1995) Forager size and ecology of *Acromyrmex coronatus* and other leaf-cutting ants in Costa Rica. Oecologia 104: 409–415. 28307655 10.1007/BF00341337

[27] Mikheyev AS, Mueller UG, Boomsma JJ (2007) Population genetic signatures of diffuse co-evolution between leaf-cutting ants and their cultivar fungi. Mol Ecol 16: 209–216 10.1111/j.1365-294X.2006.03134.x 17181732

[28] Weber NA (1969) Ecological relations of three *Atta* species in Panama. Ecology 50: 141–147.

[29] Berish C (1986) Leaf-cutting ants (*Atta cephalotes*) select nitrogen-rich forage. Am Midl Nat 115: 268–276.

[30] Cherrett JM (1972) Some factors involved in the selection of vegetable substrate by *Atta cephalotes* (L.) (Hymenoptera: Formicidae) in tropical rain forest. J Anim Ecol 41: 647–660.

[31] Farji-Brener AG (2001) Why are leaf-cutting ants more common in early secondary forests than in old-growth tropical forests? An evaluation of the palatable forage hypothesis. Oikos 92: 169–177.

[32] Howard JJ (1988) Leafcutting and diet selection: Relative influence of leaf chemistry and physical features. Ecology 69: 250–260.

[33] Mikheyev AS, Mueller UG, Abbot P (2010) Comparative dating of attine ant and lepiotaceous cultivar phylogenies reveals coevolutionary synchrony and discord. Am Nat 175: E126–E133 10.1086/652472 20415533

[34] Kooij PW, Schiøtt M, Boomsma JJ, De Fine Licht HH (2011) Rapid shifts in *Atta cephalotes* fungus-garden enzyme activity after a change in fungal substrate (Attini, Formicidae). Insect Soc 58: 145–151 10.1007/s00040-010-0127-9 PMC3059815 21475686

[35] Ibáñez R, Condit RS, Angehr G, Aguilar S, García T, et al (2002) An ecosystem report on the Panama Canal: monitoring the status of the forest communities and the watershed. Environ Monit Assess 80: 65–95. 12437064 10.1023/a:1020378926399

[36] R Core Team (2013) R: A language and environment for statistical computing. Available: http://www.R-project.org/.

[37] Pinheiro J, Bates D, DebRoy S, Sarkar D, R Core Team (2013) nlme: Linear and Nonlinear Mixed Effects Models. Available: http://cran.r-project.org/web/packages/nlme/index.html.

[38] Hothorn T, Bretz F, Westfall P (2008) Simultaneous inference in general parametric models. Biometrical J 50: 346–363. 10.1002/bimj.200810425 18481363

[39] Warnes GR, Ben Bolker, Bonebakker L, Gentleman R, Liaw WHA, et al. (2012) gplots: Various R programming tools for plotting data. Available: http://CRAN.R-project.org/package=gplots

[40] Suzuki R, Shimodaira H (2011) pvclust: Hierarchical clustering with p-values via multiscale bootstrap resampling. Available: http://CRAN.R-project.org/package=pvclust.

[41] Rønhede S, Boomsma JJ, Rosendahl S (2004) Fungal enzymes transferred by leaf-cutting ants in their fungus gardens. Mycol Res 108: 101–106 10.1017/S0953756203008931 15035511

[42] Kacuráková M, Capek P, Sasinková V, Wellner N, Ebringerová A (2000) FT-IR study of plant cell wall model compounds: pectic polysaccharides and hemicelluloses. Carbohyd Polym 43: 195–203 10.1016/S0144-8617(00)00151-X

[43] Husson F, Josse J, Le S, Mazet J (2013) FactoMineR: Multivariate Exploratory Data Analysis and Data Mining with R. Available: http://CRAN.R-project.org/package=FactoMineR.

[44] De Vasconcelos HL (1990) Foraging activity of two species of leaf-cutting ants (Atta) in a primary forest of the central amazon. Insect Soc 37: 131–145 10.1007/BF02224026

[45] Villesen P, Murakami T, Schultz TR, Boomsma JJ (2002) Identifying the transition between single and multiple mating of queens in fungus-growing ants. P R Soc B 269: 1541–1548 10.1098/rspb.2002.2044 PMC1691065 12184823

[46] Murakami T, Higashi S, Windsor D (2000) Mating frequency, colony size, polyethism and sex ratio in fungus-growing ants (Attini). Behav Ecol Sociobiol 48: 276–284.

[47] Littledyke M, Cherrett JM (1976) Direct ingestion of plant sap from cut leaves by the leaf-cutting ants *Atta cephalotes* (L.) and *Acromyrmex octospinosus* (Reich) (Formicidae, Attini). B Entomol Res 66: 205–217.

[48] Quinlan R, Cherrett JM (1979) The role of fungus in the diet of the leaf-cutting ant *Atta cephalotes* (L.). Ecol Entomol 4: 151–160.

[49] Mendes TD, Rodrigues A, Dayo-Owoyemi I, Marson FAL, Pagnocca FC (2012) Generation of nutrients and detoxification: possible roles of yeasts in leaf-cutting ant nests. Insects 3: 228–245 10.3390/insects3010228 26467957 PMC4553625

[50] Suen G, Scott JJ, Aylward FO, Adams SM, Tringe SG, et al. (2010) An insect herbivore microbiome with high plant biomass-degrading capacity. PLoS Genet 6. doi:10.1371/journal.pgen.1001129. PMC2944797 20885794

[51] North RD, Jackson CW, Howse PE (1999) Communication between the fungus garden and workers of the leaf-cutting ant, *Atta sexdens rubropilosa*, regarding choice of substrate for the fungus. Physiol Entomol 24: 127–133 10.1046/j.1365-3032.1999.00122.x

[52] Aylward FO, Burnum-Johnson KE, Tringe SG, Teiling C, Tremmel DM, et al (2013) *Leucoagaricus gongylophorus* produces diverse enzymes for the degradation of recalcitrant plant polymers in leaf-cutter ant fungus gardens. Appl Environ Microb 79: 3770–3778 10.1128/AEM.03833-12 PMC3675943 23584789

[53] Kooij PW (unpublished) Fungal adaptations to mutualistic life with ants. Copenhagen: Copenhagen University. 156 pp.

[54] Bot A, Currie CR, Hart AG, Boomsma JJ (2001) Waste management in leaf-cutting ants. Ethol Ecol Evol 13: 225–237.

[55] Hart AG, Ratnieks FLW (2002) Waste management in the leaf-cutting ant *Atta colombica* . Behav Ecol 13: 224–231.

[56] Moller IE, De Fine Licht HH, Harholt J, Willats WGT, Boomsma JJ (2011) The dynamics of plant cell-wall polysaccharide decomposition in leaf-cutting ant fungus gardens. PLoS ONE 6: e17506 10.1371/journal.pone.0017506 21423735 PMC3053354

[57] Nagamoto NS, Garcia MG, Forti LC, Verza SS, Noronha NC, et al (2011) Microscopic evidence supports the hypothesis of high cellulose degradation capacity by the symbiotic fungus of leaf-cutting ants. J Biol Res-Thessalon 16: 308–312.

[58] Grell MN, Nielsen KL, Linde T, Nygaard S, Boomsma JJ, et al (2013) The fungal symbiont of leaf-cutter ants expresses the full enzyme complement to degrade cellulose and other plant polymers. BMC Genomics 14: 928 10.1186/1471-2164-14-928 24373541 PMC3880420

[59] Tyrrell HF, Moe PW (1975) Effect of intake on digestive efficiency. J Dairy Sci 58: 1151–1163.

[60] Porter SD, Bowers MA (1980) Emigration of an *Atta* colony. Biotropica 12: 232–233.

[61] Crewe RM, Blum MS (1972) Alarm pheromones of the Attini: their phylogenetic significance. J Insect Physiol 18: 31–42.

[62] Wetterer JK, Gruner D, Lopez J (1998) Foraging and nesting ecology of *Acromyrmex octospinosus* (Hymenoptera : Formicidae) in a Costa Rican tropical dry forest. Fla Entomol 81: 61–67.

